# Prehypertension and Endothelial Dysfunction: The Role of Oxidative Stress

**DOI:** 10.3390/biomedicines14102220

**Published:** 2026-10-01

**Authors:** Ivo Vincetić, Ana Stupin, Ivana Tolj, Ivana Jukić, Ines Drenjančević, Sandra Šarić, Marko Vincetić, Marin Vučković, Marko Stupin

**Affiliations:** 1 Department of Radiology, University Hospital Center Osijek, 31000 Osijek, Croatia; ivovincetic5@gmail.com; 2 Department of Physiology and Immunology, Faculty of Medicine Osijek, Josip Juraj Strossmayer University of Osijek, 31000 Osijek, Croatia; ana.stupin@mefos.hr (A.S.); ivana.jukic@mefos.hr (I.J.); 3 Department of Internal Medicine, Faculty of Medicine Osijek, Josip Juraj Strossmayer University of Osijek, 31000 Osijek, Croatia; ivanatolj5@gmail.com; 4 Department of Nephrology, University Hospital Center Osijek, 31000 Osijek, Croatia; 5 Department of Cardiovascular Disease, University Hospital Center Osijek, 31000 Osijek, Croatia; ssaric@mefos.hr (S.Š.); markovincetic1999@gmail.com (M.V.); marin.vuckovic@gmail.com (M.V.); 6 Department of Pathophysiology, Faculty of Medicine Osijek, Josip Juraj Strossmayer University of Osijek, 31000 Osijek, Croatia

**Keywords:** prehypertension, endothelial dysfunction, oxidative stress, vascular dysfunction, arterial stiffness, microvascular alterations, nitric oxide, cardiovascular prevention

## Abstract

Prehypertension, defined as blood pressure of 120–139/80–89 mmHg, affects approximately 36–38% of adults worldwide and is associated with a substantially increased risk of cardiovascular disease, stroke, and coronary heart disease. Beyond being a transitional stage toward overt hypertension, prehypertension represents a biologically active vascular state in which early vascular injury is already evident. Endothelial dysfunction is considered the earliest detectable vascular abnormality in prehypertension and is characterized by reduced nitric oxide bioavailability, endothelial nitric oxide synthase (eNOS) uncoupling, and increased asymmetric dimethylarginine levels. These alterations are accompanied by endothelial activation, reflected by enhanced expression of adhesion molecules and endothelin-1, as well as impaired endothelial progenitor cell function with a reported threshold effect at systolic blood pressure of 130 mmHg. In parallel, prehypertensive individuals exhibit increased arterial stiffness and microvascular abnormalities, including systemic capillary rarefaction and retinal microvascular alterations. Oxidative stress, driven mainly by NADPH oxidase activation and eNOS uncoupling, appears to be a central mechanism linking elevated blood pressure with endothelial dysfunction and progressive vascular damage. However, the temporal relationship between oxidative imbalance, endothelial dysfunction, and blood pressure progression remains unclear. It is still uncertain whether oxidative stress initiates vascular impairment or primarily accelerates already established endothelial injury. Further prospective studies are needed to define the vascular trajectory of prehypertension, clarify its prognostic relevance, and identify whether early vascular alterations represent therapeutic targets for cardiovascular prevention. By integrating current evidence, this review examines endothelial dysfunction in prehypertension, with particular attention to oxidative stress and its interactions with NO signaling, endothelial activation, and vascular remodeling.

## 1. Introduction

The concept of prehypertension was introduced by the Seventh Report of the Joint National Committee on Prevention, Detection, Evaluation, and Treatment of High Blood Pressure in 2003 (JNC 7) for those individuals with blood pressures ranging from 120 to 139 mm Hg systolic (SBP) and/or 80 to 89 mm Hg diastolic blood pressure (DBP) [1]. This classification was based on meta-analyses by the Prospective Studies Collaboration which showed a sustained, proportional link between blood pressure and mortality risk starting from levels as low as 115/75 mmHg [2]. The American College of Cardiology/American Heart Association (ACC/AHA) guidelines from 2017 abandoned the term prehypertension [3], and the revised scheme uses four bands: normal blood pressure (<120/80), elevated blood pressure (120–129/<80), stage 1 hypertension (130–139/80–89), and stage 2 hypertension (≥140/90). Due to this reclassification most individuals previously classified as prehypertensive were reclassified as having stage 1 hypertension. Subsequently, this reclassification increased prevalence of hypertension from 32% to 46% among US adults and added approximately 31 million newly diagnosed hypertensives [4].

The latest ESH guidelines from 2023 maintained a more conservative approach with classifying blood pressure ranges into optimal (<120/80 mmHg), normal (120–129/80–84 mmHg), high-normal (130–139/85–89 mmHg), and grade 1 hypertension (starting at 140/90 mmHg) was retained. The category that functionally corresponds to prehypertension is high-normal blood pressure [5], defined as systolic blood pressure of 130–139 mmHg and/or diastolic blood pressure of 85–89 [5]. This difference in thresholds is based on different approaches to hypertension by the American (ACC/AHA) and European (ESC/ESH) cardiology societies; while the ACC/AHA guidelines place primary emphasis on cardiovascular risk, the ESC/ESH guidelines prioritize treatment benefit at specific thresholds [6].

Prehypertension is associated with increased cardiovascular risk and affects the cardiovascular health of the population [7,8]. Data show that prehypertension affects 36–38% of adults worldwide increasing the risk of cardiovascular diseases by 55%, stroke by 71%, and coronary heart disease by 50% [9,10,11]. Thus, prehypertension is not just a blood pressure classification but represents a critical period in which vascular dysfunction already begins before overt hypertension develops.

Individuals with prehypertension show clear signs of endothelial dysfunction. Studies have shown that endothelial dysfunction in prehypertension is characterized by approximately a 30% lower acetylcholine-induced forearm vasodilation [7], increased oxidative stress markers [12], a 47% decline in endothelial progenitor cell (EPC) repair capacity [13], and greater arterial stiffness [8]. The changes caused by prehypertension are not just consequences of elevated blood pressure, but they are an indicator of active pathophysiological processes. These processes begin with endothelial damage driven by oxidative stress [12].

Effective prevention strategies could benefit roughly 25–50% of adults worldwide with prehypertension who are at increased risk of cardiovascular disease [14]. To develop useful prevention strategies, it is important to better understand the pathophysiological mechanisms behind prehypertension and endothelial dysfunction. Thus, the aim of this review is to examine endothelial dysfunction in prehypertension, with particular attention to oxidative stress and its interactions with NO signaling, endothelial activation, and vascular remodeling.

## 2. Global Prevalence and Cardiovascular Risk Burden

Epidemiological studies report that prehypertension, as defined by JNC 7, affects approximately 36–38% of adults worldwide and is more common in men than in women [7,9,15]. Prevalence estimates vary substantially across studies because blood pressure categories and thresholds differ [16,17,18]. Prehypertension also frequently progresses to overt hypertension: in the Framingham Heart Study, the four-year progression risk was higher among individuals in the upper than in the lower prehypertensive range (43% vs. 20%) [19,20].

This early elevation in blood pressure is associated with increased cardiovascular risk. A meta-analysis of 18 prospective cohorts involving 468,561 participants found an increased risk of cardiovascular disease (RR 1.55; 95% CI: 1.41–1.71), stroke (RR 1.71; 95% CI: 1.55–1.89), and coronary heart disease (RR 1.50; 95% CI: 1.30–1.74) among individuals with prehypertension. Risk was elevated even in the lower prehypertensive range and was greater in the upper range [11]. These findings provide a clinical rationale for investigating vascular changes before established hypertension develops.

## 3. Endothelial Dysfunction as an Early Event in Prehypertension

Endothelial dysfunction has been suggested as the earliest detectable vascular abnormality in prehypertension, preceding structural vascular changes and established hypertension [21,22,23]. Similar to the hypothesis that endothelial dysfunction develops during the prehypertensive stage and precedes the onset of overt arterial hypertension, comparable pathophysiological mechanisms have been described in the context of high dietary salt intake. Beyond its established role in raising blood pressure, salt intake may also directly impair vascular function. A growing body of work shows that high salt intake induces endothelial dysfunction independently of any change in blood pressure. Studies in young, healthy, normotensive individuals, have repeatedly shown that a seven-day high-salt diet impairs microvascular and brachial-artery endothelium-dependent vasodilation without change in blood pressure, body composition, or body fluid status. These data indicate a direct, pressure-independent effect of salt on the endothelium [24,25,26]. In studies using human and animal models of short-term salt loading, this impairment of micro-and macrovascular endothelium-dependent vasodilation is mechanistically linked to increased vascular oxidative stress and reduced NO bioavailability [27,28]. It is important to emphasize that high salt intake also promotes a pro-inflammatory vascular phenotype. In healthy individuals, short-term salt loading initiates low-grade vascular inflammation [29]. Likewise, a short-term high-sodium diet alters leukocyte expression of the cell adhesion molecules—the integrins VLA-4, LFA-1, and Mac-1—in both humans and rats [30]. A further line of evidence supporting oxidative stress as the central mechanism comes from antioxidant studies: enhanced antioxidant defense with vitamins C and E prevents this salt-induced microvascular impairment [24]. Together, these findings indicate that dietary salt is an independent contributor to endothelial dysfunction and vascular inflammation—mechanisms that overlap with those underlying prehypertension and may aggravate vascular risk even before blood pressure rises.

## 4. Mechanisms of Endothelial Dysfunction in Prehypertension

Among many potential mechanisms that have been involved in endothelium-dependent vascular reactivity, the central mechanism of endothelial dysfunction revolves around the balance between NO and reactive oxygen species, which transforms the endothelium condition from a protective, anti-thrombotic, and anti-inflammatory into an activated, pro-thrombotic, and pro-inflammatory state [22,31]. Most studies investigating vascular alterations in prehypertension have therefore focused on endothelial nitric oxide (NO) bioavailability, given its central role in maintaining vascular homeostasis.

In prehypertension, NO bioavailability is significantly reduced through several interrelated mechanisms. For example, prehypertensive subjects exhibited approximately 30% reduction in NO-mediated vasodilation compared to normotensive controls in forearm resistance (microvascular) vessels, assessed by venous occlusion plethysmography during intra-arterial acetylcholine infusion. Administration of acetylcholine, an endothelium-dependent vasodilator, increased forearm blood flow from 4.2 ± 0.3 to 11.4 ± 0.7 mL/100 mL tissue/min in prehypertensives compared to 4.6 ± 0.2 to 14.5 ± 0.7 mL/100 mL tissue/min in normotensives [7]. Inhibition of NOS with its competitive inhibitor L-NMMA significantly reduced acetylcholine-mediated vasodilation in normotensives by approximately 30%, but no effect was observed in prehypertensives, indicating that NO-dependent pathways in prehypertension have been impaired already [7].

The concept of endothelial NO synthase (eNOS) uncoupling is crucial for understanding oxidative stress in prehypertension [32,33]. Under normal physiological conditions, eNOS functions as a homodimer and requires L-arginine, oxygen, NADPH, and the essential cofactor tetrahydrobiopterin (BH4) for normal activity. When BH4 is oxidized to dihydrobiopterin (BH2) by peroxynitrite, or when L-arginine levels are reduced, eNOS undergoes conformational uncoupling. The uncoupled enzyme then produces superoxide radicals instead of NO [34]. This change turns eNOS from a protective enzyme into a pathogenic source of reactive oxygen species (ROS), which further increases the initial oxidative stress generated by NADPH oxidase and mitochondrial sources [12,33]. Two lines of evidence support this mechanism in hypertensive vasculature: loss of eNOS function reduces vascular superoxide in a hypertensive model, and BH4 supplementation restores coupling, improves endothelial function, and reduces the rise in blood pressure [35]. In animal models eNOS uncoupling occurs already in the prehypertensive stage: in aortas of 4-week-old, still-normotensive spontaneously hypertensive rats NOS increases superoxide production and decreases NO production with L-NMMA inhibiting ~70% of this superoxide [32]. However, there is no direct evidence of eNOS uncoupling and BH4 oxidation in prehypertensive human studies and it remains to be proven.

Asymmetric dimethylarginine (ADMA) is a methylated derivative of the amino acid L-arginine. ADMA has been proposed to be a key regulator of the NO/ROS balance in the development of hypertension, including the prehypertensive stage [36]. ADMA also competitively inhibits both constitutive and inducible isoforms of nitric oxide synthase (NOS) [37]. Its pathogenetic role is twofold: in addition to reducing NO production, ADMA simultaneously stimulates eNOS toward an “uncoupled” state and source of superoxide anion instead of NO [38]. On the synthesis side, ADMA synthesis depends on the enzyme protein-arginine methyltransferase type I (PRMT-1), whose expression and activity increase during oxidative stress. Conversely, ADMA is degraded by dimethylarginine dimethylaminohydrolase 1 and 2 (DDAH-1 and DDAH-2), which are inhibited by ROS [37]. This mechanism creates a vicious cycle in the context of oxidative stress by simultaneously increasing ADMA synthesis and decreasing its degradation, leading to the accumulation of excess ADMA [39]. In patients with essential hypertension, elevated plasma ADMA levels have been verified, where ADMA increases progressively from the prehypertensive range (120–139/80–89 mmHg) through stage 1 and stage 2 of hypertension, and is independently correlated with systolic blood pressure [40]. Direct human evidence specifically in prehypertension remains limited, however, and the relationship between circulating ADMA and blood pressure in non-hypertensive individuals has been inconsistent.

**Figure 1:**
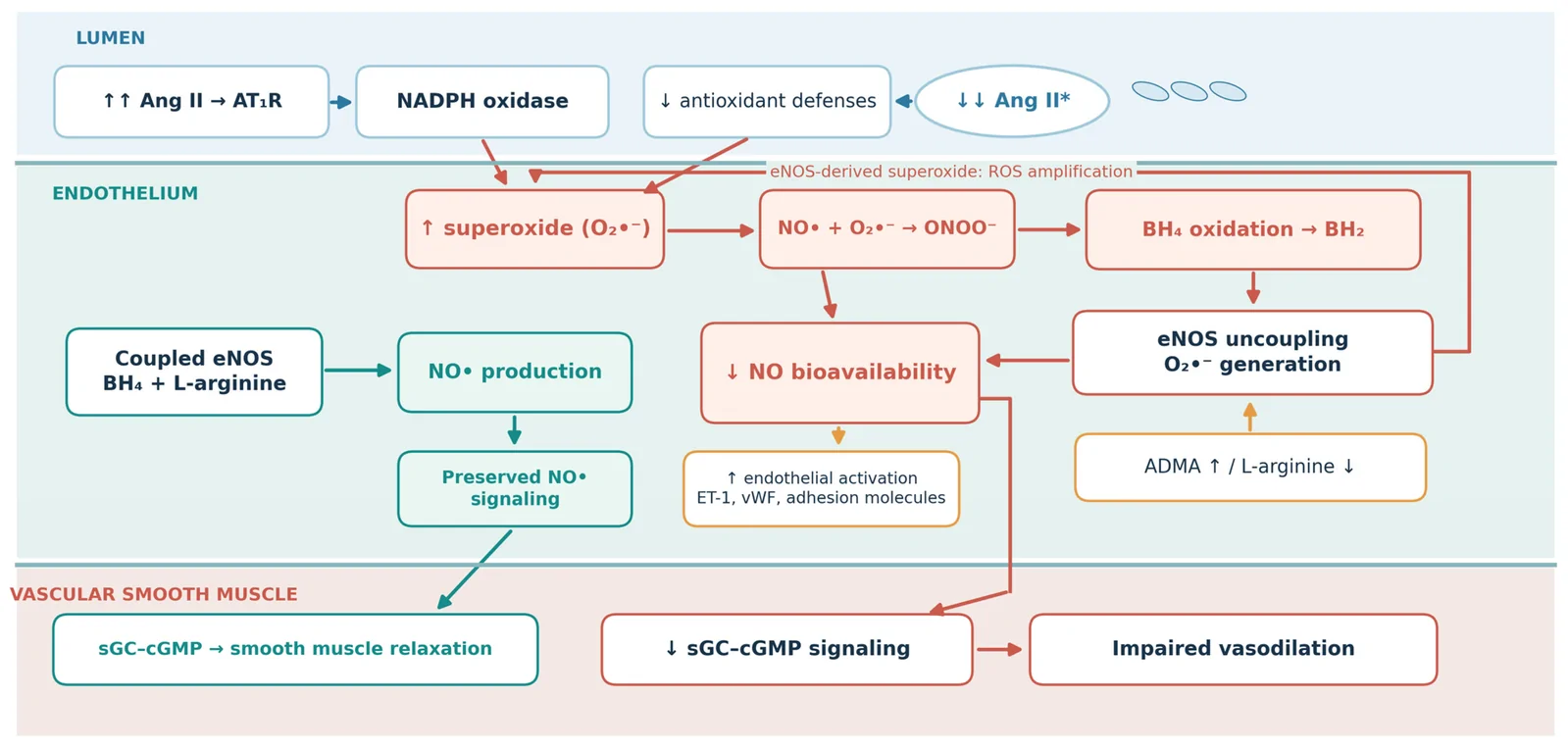
Interacting mechanisms of oxidative stress and endothelial dysfunction in prehypertension. Angiotensin II–AT_1_ receptor signaling and vascular NADPH oxidases may increase superoxide production, while reduced antioxidant defenses may limit its removal. Superoxide decreases nitric oxide (NO) bioavailability and contributes to peroxynitrite formation. Oxidation of tetrahydrobiopterin (BH_4_) to dihydrobiopterin (BH_2_), together with reduced L-arginine availability or increased asymmetric dimethylarginine (ADMA), can promote endothelial NO synthase (eNOS) uncoupling. Uncoupled eNOS generates additional superoxide instead of NO, creating a self-amplifying cycle. Reduced NO signaling through soluble guanylate cyclase (sGC) and cyclic guanosine monophosphate (cGMP) impairs vascular smooth muscle relaxation and favors endothelial activation. The diagram summarizes biologically plausible interactions; their temporal sequence and causal contribution in human prehypertension remain to be established. Solid arrows: proposed interactions; sequence and causality in human prehypertension remain uncertain. Upward arrows (↑) indicate an increase, whereas downward arrows (↓) indicate a decrease. Double arrows (↑↑ or ↓↓) denote markedly increased or decreased angiotensin II (Ang II) levels, respectively. These symbols are qualitative and do not represent quantified changes. * Low ANG II pathway: findings from specific experimental settings; not established as a general mechanism in human prehypertension.

Taken together, these findings show how changes in NO signaling, eNOS activity, ADMA, and endothelial activation may reinforce one another. Oxidative stress intersects with several of these pathways, but the extent to which it initiates rather than amplifies endothelial dysfunction in human prehypertension remains uncertain. Its potential contribution is considered in more detail in Section 9. The interacting pathways linking oxidative imbalance, eNOS uncoupling, and impaired NO signaling are summarized in Figure 1.

## 5. Biomarkers of Endothelial Activation and Dysfunction

NO inhibits NF-κB–driven expression of adhesion molecules and thereby suppresses endothelial activation. The deficiency in NO bioavailability that characterizes the prehypertensive endothelium drives endothelial activation toward a pro-adhesive and pro-inflammatory state [41]. The transition from a quiescent to an activated endothelial state in hypertension is marked by changes in adhesion molecules, produced by activated endothelial cells, which represent biomarkers of endothelial activation and dysfunction. Intercellular adhesion molecule-1 (ICAM-1) is significantly elevated in essential hypertension (238 ng/mL) compared to normotensive individuals (208 ng/mL, *p* = 0.02), thereby mediating leukocyte transmigration during vascular inflammation [42]. In the Framingham Offspring/Generation-3 study of 5405 adults free of prior cardiovascular disease, the endothelial adhesion molecules ICAM-1 and P-selectin rose progressively across ascending AHA/ACC blood pressure categories (trend *p* < 0.0001) [43]. Both were already higher in the Elevated category (120–129/<80) than in normotensive individuals [43]. Shedding of these adhesion molecules may serve as a marker of endothelial activation early in the course of rising blood pressure. Vascular cell adhesion molecule-1 (VCAM-1), expressed on activated endothelial cells, is similarly elevated in essential hypertension [44,45] and, unlike ICAM-1, its expression precedes lesion formation and is selectively induced during early atherogenesis [46,47]. VCAM-1 interacts with the integrin VLA-4 (α4β1) on monocytes and lymphocytes to promote their firm adhesion to the activated endothelium [48]. Whether circulating VCAM-1 are elevated in prehypertension remains to be established. However, increased expression of these adhesion molecules can be viewed as an indicator of an imbalance in the NO/ROS system. As NO falls and ROS rises, the endothelium transitions into an activated surface that actively recruits leukocytes.

Endothelin-1 (ET-1), the most potent endogenous vasoconstrictive peptide, which is released from endothelium upon the injury of blood vessel. It binds to two G-protein–coupled receptors: ETA receptors and ETB receptors. ETA receptors are located predominantly on vascular smooth muscle cells and mediate vasoconstriction, whereas ETB receptors mediate vasodilation through the release of NO and prostacyclin and are localized mainly on endothelial cells [49]. ET-1 has been significantly elevated in essential hypertension compared to normotensive individuals [42]. Importantly, functional studies indicate that the use of the selective endothelin-A (ETA) receptor antagonist BQ-123 in prehypertensive adults causes an approximately 20% increase in forearm blood flow in response to ETA receptor blockade, compared to an increase of only 5% in normotensive individuals. These data suggest that ET-1 causes enhanced vasoconstriction in prehypertension and contributes to endothelial dysfunction even in the early stage of the disease [50]. ET-1 and NO mutually regulate each other and are maintained in balance [49]. NO suppresses ET-1 expression [49], while ET-1 promotes superoxide production [51]. In the prehypertensive endothelium, NO bioavailability falls and consequently ET-1 rises, which together with increased superoxide could lead to a NO/ROS imbalance [49,51].

Vascular injury and endothelial activation also lead to increased secretion of von Willebrand factor (vWF), produced mainly by the endothelium. It is well known that NO regulates and restrains the process of vWF secretion. NO from the endothelium inhibits the exocytosis of Weibel–Palade bodies—the endothelial granules that store vWF—through S-nitrosylation of N-ethylmaleimide–sensitive factor (NSF) [52]. Furthermore, pharmacological inhibition of NO synthase increases stimulated vWF release in healthy individuals [53]. Likewise, reduced NO synthesis in endothelial dysfunction is thought to permit enhanced WPB exocytosis, contributing to the prothrombotic, proinflammatory endothelial phenotype associated with cardiovascular risk [54]. The reduction in NO bioavailability, which also occurs in the prehypertensive endothelium, favors enhanced vWF secretion and thereby links this endothelial factor to the NO/ROS imbalance in endothelial dysfunction. vWF has been increased in essential hypertension (119 IU/dL) compared to normotensive individuals (105 IU/dL, *p* < 0.05) [42], but there are currently no specific data for prehypertension. Interestingly, gene silencing studies in porcine aortic endothelial cells suggest a functional regulatory link between vWF and ET-1, as siRNA knockdown of vWF prevents angiotensin II from upregulating ET-1 levels—an interaction worth of exploring in future studies [55].

## 6. Arterial Stiffness: Early Structural and Functional Changes

Arterial stiffness in prehypertension is mechanistically linked to endothelial imbalance. Reduced NO bioavailability in prehypertensive individuals leads to a diminished vasodilatory effect on the vascular wall. On the other hand, an excess of ET-1 promotes constriction and hypertrophy of vascular smooth muscle. ROS further amplifies this effect by scavenging NO [56]. These changes lead to stiffening of the arterial wall, and the resulting rise in central pressure and pulsatile load feeds back on the endothelium.

Therefore, in prehypertension, arterial stiffness and blood pressure may form a bidirectional vicious cycle that can accelerate progression toward hypertension and cardiovascular disease. Arterial stiffness can be understood as both a consequence of prehypertension and a contributor to its progression to hypertension [57]. Aortic PWV, measured by the carotid-femoral method, is the gold-standard measure of arterial stiffness. In prehypertensive individuals (brachial SBP 130 ± 6/DBP 84 ± 3 mm Hg), aortic PWV is significantly elevated at 10.0 ± 2.5 m/s compared with 8.6 ± 1.7 m/s in normotensive controls (106 ± 10/66 ± 10 mm Hg; *p* = 0.004) [58]. A matched case–control study of 217 untreated prehypertensive individuals (SBP 120–139/DBP 80–89 mmHg) and 217 age- and sex-matched normotensive controls (SBP < 120/DBP < 80 mmHg) found aortic PWV significantly higher in the prehypertensive group (6.13 ± 1.08 vs. 5.56 ± 0.99 m/s, *p* < 0.0001) [8].

The prognostic significance of arterial stiffness in prehypertension is considerable. A Finnish cohort study, which examined 1449 adults aged 30–45 years (1183 normotensive at baseline), found that baseline PWV was an independent predictor of incident hypertension four years later, with an odds ratio of 1.96 per 1-SD increase in PWV (95% CI: 1.51–2.57, *p* < 0.001) [59]. When stricter blood pressure thresholds were applied (SBP ≥ 120 or DBP ≥ 80 mmHg), PWV became the highest predictor, with an odds ratio of 2.16 per 1-SD increase [59]. A meta-analysis showed that higher arterial stiffness is associated with incident hypertension with a relative risk of 1.09 (95% CI: 1.05–1.12) [60], and prehypertension with increased PWV is independently associated with target organ damage, including left ventricular hypertrophy and albuminuria [61].

## 7. Flow Mediated Dilation of Brachial Artery: Large Vessels Endothelial Function

Studies evaluating flow-mediated dilation (FMD) of brachial artery (gold standard for assessment of large arteries endothelial function) indicate a gradual decline in vascular function progressing from normotensive states through prehypertension and toward established hypertension—although the evidence at each stage derives from separate populations rather than a single cohort followed across the continuum. In a cross-sectional study of healthy adults free of other risk factors, in which subjects were stratified by systolic blood pressure in 10-mmHg increments, FMD declined progressively as pressure rose across the 115–139 mmHg range (16.2 ± 5.6%, 13.4 ± 5.2%, and 11.5 ± 3.6% in ascending strata; *p* < 0.05). By contrast, the endothelium-independent (nitrate-mediated) response did not differ significantly across strata (*p* = 0.217), indicating that the decline was endothelium-specific [62]. Young individuals with prehypertension (SBP 120–139 mmHg and DBP 80–89 mmHg) show a 26–36% decrease in flow-mediated dilation (FMD) compared to those with normal blood pressure [63]. Separately, in middle-aged individuals with established hypertension FMD decreases to 5.4 ± 2.8%, compared to 6.9 ± 3.1% in those without hypertension (*p* < 0.001) [64]. The studies cited were conducted using different measurement protocols and in different populations, but the consistent finding is the direction of change—progressively lower endothelial function with rising arterial pressure.

## 8. Microcirculatory Dysfunction: The Small Vessel Contribution

While arterial stiffness and impaired FMD affect large conducting blood vessels, prehypertension is also associated with significant changes in the microcirculation that may precede and contribute to increased blood pressure through higher peripheral resistance [65]. Capillary rarefaction, defined as a decrease in capillary density, initially appears as a functional disorder with reduced perfusion of existing capillary networks and may progress to structural rarefaction, characterized by actual regression and loss of capillary blood vessels [65]. Importantly, research on elevated blood pressure states (SBP 130–159 mmHg compared with normotensive levels SBP < 130 mmHg) shows the presence of functional capillary rarefaction in the absence of structural loss, indicating an early alteration [65], that appears reversible with effective antihypertensive treatment in low-risk hypertensive patients [66]. In cases of confirmed hypertension, skin (video) capillaroscopy demonstrates a capillary density of 71.3 ± 1.5 capillaries/mm^2^, significantly lower than the 80.6 ± 1.8 capillaries/mm^2^ observed in normotensive individuals (*p* < 0.001), indicating an approximate 12% decrease [66].

Prehypertensive microvascular dysfunction has not been adequately studied, especially regarding functional assessments of the peripheral microcirculation. The existing evidence is limited and largely based on coronary circulation studies, in which prehypertensive individuals have had a significantly lower coronary flow reserve (CFR 2.54 ± 0.48 compared with 2.91 ± 0.53 in normotensive individuals, *p* < 0.01), indicating that microvascular dysfunction may already be detectable at this early stage of elevated blood pressure [67]. The Rotterdam study on retinal vessel diameters and hypertension found that each standard deviation decrease in arteriolar diameter is associated with a 38% higher risk of developing hypertension (OR 1.38; 95% CI 1.23–1.55) [68]. It is important to emphasize that narrowing of the arterioles can already be seen in the prehypertensive stage, as adolescents with prehypertension have Central Retinal Arteriolar Equivalent (CRAE) values similar to those with hypertension and significantly lower than those of normotensive individuals [69]. For example, in patients without coronary calcification, myocardial perfusion reserve declined progressively with narrower retinal arterioles (3.52, 3.37, 3.19, 3.10; *p* = 0.01). These findings suggest that changes in retinal arterioles may reflect broader systemic microvascular impairment, also detectable in the coronary microcirculation [70].

**Figure 2:**
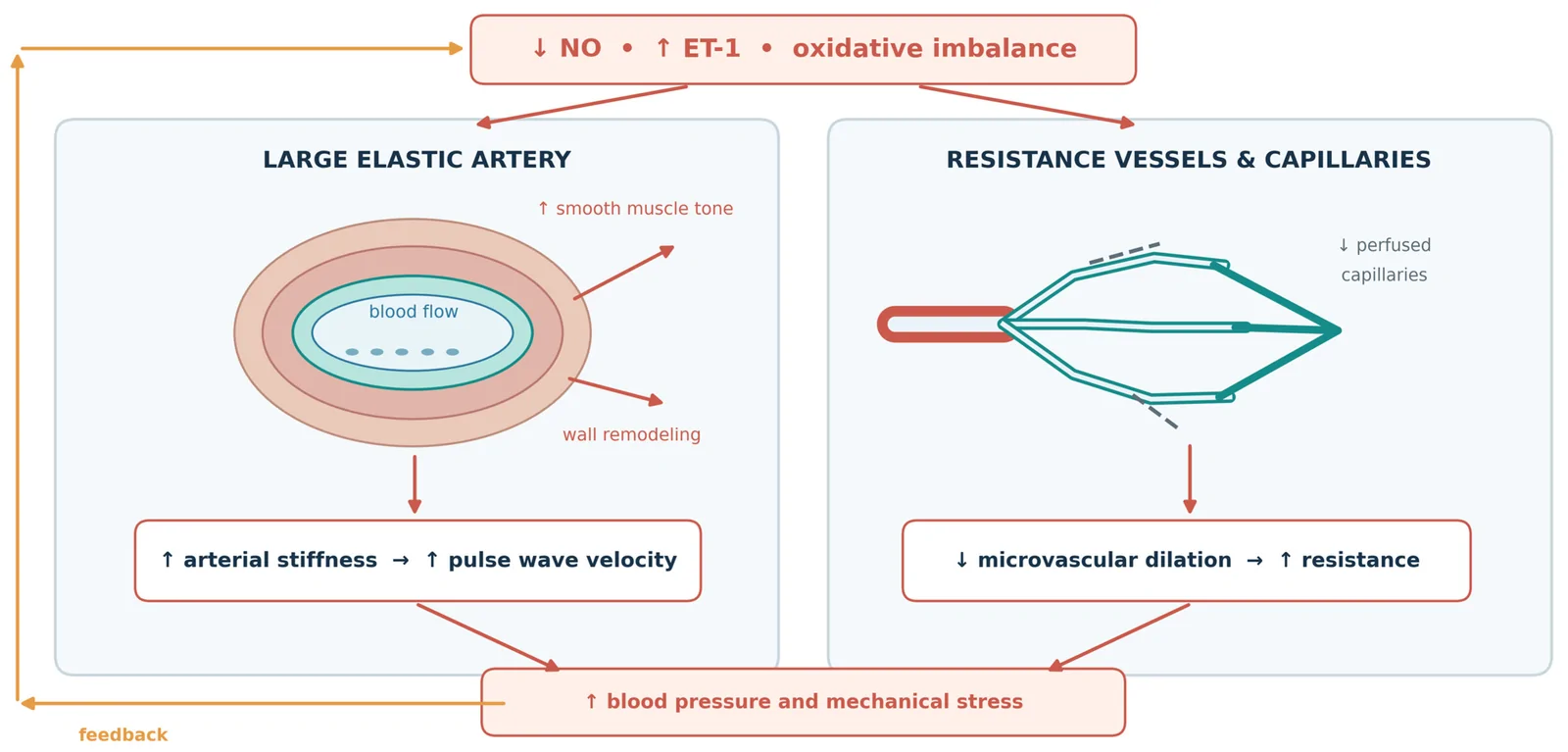
Proposed links between endothelial dysfunction, arterial stiffness, and microvascular alterations in prehypertension. Reduced NO bioavailability and increased endothelin-1 (ET-1) activity may shift vascular tone toward vasoconstriction and promote changes in vascular smooth muscle. In large arteries, these processes may contribute to increased arterial stiffness, faster pulse wave propagation, and greater central pulsatile load. In resistance vessels and the microcirculation, impaired endothelium-dependent dilation and reduced capillary perfusion may increase peripheral vascular resistance. Higher blood pressure and pulsatile stress may, in turn, aggravate endothelial dysfunction. Dashed gray lines schematically represent non-perfused capillary segments (functional rarefaction), rather than confirmed structural loss of capillaries. The arrows depict proposed, potentially bidirectional relationships rather than a proven sequence of events in people with prehypertension.

The previously mentioned study, using venous occlusion plethysmography during intra-arterial acetylcholine infusion, demonstrated reduced NO-mediated vasodilation in prehypertensive individuals, shown by a smaller increase in forearm blood flow in response to acetylcholine by the fact that NOS inhibition with L-NMMA—which reduced the response in normotensives—had little effect in prehypertensives [7]. Studies involving established cases of essential hypertension have shown a clear reduction in skin microvascular function measured by laser Doppler flowmetry, with lower post-occlusive reactive hyperemia (PORH) peak flow (287 ± 31.5% of baseline compared with 410 ± 35% in normotensive individuals, *p* < 0.01) [71]. Similarly, endothelium-dependent acetylcholine-induced vasodilation was significantly reduced in essential hypertension compared with normotensive controls, both at low doses (206 ± 34% vs. 444 ± 80%, *p* < 0.01) and at high doses (569 ± 54% vs. 804 ± 93%, *p* < 0.05) [71]. These results suggest significant peripheral microvascular dysfunction in established hypertension and also point out the limited availability of comparable non-invasive functional data on peripheral microcirculation in prehypertension. Figure 2 illustrates how endothelial dysfunction may affect large arteries and the microcirculation, with potential feedback from increased blood pressure.

## 9. Oxidative Stress and Its Interaction with Endothelial Dysfunction

The endothelial changes discussed above may be linked, in part, by an imbalance between reactive oxygen species (ROS) production and antioxidant defenses [72,73]. Increased ROS can reduce NO bioavailability, while impaired NO signaling and eNOS uncoupling may further contribute to oxidative imbalance. These interactions provide a rationale for examining oxidative stress alongside, rather than as an established cause of, endothelial dysfunction in prehypertension. For example, antioxidant enzyme activity declines as early as the prehypertensive phase, before any significant rise in lipid peroxidation can be detected [74].

NADPH oxidase is a major enzymatic source of superoxide in the vasculature of hypertensive individuals [75]. Experimental findings in the spontaneously hypertensive rats model showed that NADPH oxidase subunit p47phox was significantly upregulated during the prehypertensive phase, before blood pressure began to rise [76]. Various Nox isoforms contribute in a cell-specific manner. Nox1 is predominantly expressed in vascular smooth muscle, where it amplifies angiotensin II–induced hypertension and vascular superoxide production [77]. Nox2 (gp91phox), the dominant isoform in endothelial cells, is upregulated in hypertension [78]. On the other hand, the loss of Nox4 promotes endothelial dysfunction owing to its vasoprotective action via the production of hydrogen peroxide [79,80]. Subunits such as p47phox and p22phox are required for assembly and activity of the vascular NADPH oxidase. Studies show that genetic knockout or silencing of either subunit significantly reduces the blood pressure response to angiotensin II [81,82].

Importantly, NADPH oxidase is activated by angiotensin II via AT_1_ receptor (AT_1_R) signaling. This pathway directly connects the renin–angiotensin system and oxidative stress [83]. And, although increased angiotensin II levels are well recognized to promote oxidative stress, experimental data from animal and human controlled studies demonstrate that decreased or low- non modulated levels of angiotensin II are also linked to increased oxidative stress due to impaired antioxidative system activation [84,85]. This has been shown in genetically modified rat strains but also in animals and human subjects on a high salt diet [26,86,87,88]. These findings suggest that physiological angiotensin II signaling is required for normal vascular function, whereas both excessive activation and excessive suppression of the renin–angiotensin system may contribute to oxidative stress.

Similarly, in the experimental model, chronic blockade of AT_1_R leads to increase in ROS and decrease in NO [89]. In healthy cerebral arteries, tonic AT_1_R activation by physiological angiotensin II maintains the mechanism of NO-mediated dilation and the balance between oxidants and antioxidants. Chronic AT_1_R blockade with losartan increased vascular and systemic oxidative stress and reduced NO bioavailability. The superoxide scavenger TEMPOL restored dilation and its NO-dependent component [89]. Thus, interplay of Ang II and endothelial dysfunction is very complex and endothelium-mediated vascular reactivity is dependent on physiological levels of angiotensin II. In prehypertension, direct data on circulating angiotensin II are lacking. Plasma angiotensin II levels are unlikely to capture the relevant signal: angiotensin II correlates poorly with blood pressure status even in established hypertension. Likewise, endothelial effects are governed largely by the local vascular renin–angiotensin system rather than by circulating concentrations.

Uncoupled eNOS amplifies oxidative stress by increasing ROS production through BH4 oxidation and therefore reducing NO bioavailability even further [90]. This concept is supported by evidence showing that blocking eNOS leads to a paradoxical decrease in vascular superoxide in hypertensive vessels. In addition, BH4 supplementation reduces eNOS-derived superoxide production and restores vascular NO production, thereby normalizing the mechanism of vasodilation and blunting the rise in blood pressure in this model [35].

Oxidative stress reduces the bioavailability of NO in two ways. First, superoxide directly “binds” and neutralizes NO, faster than superoxide dismutase (SOD) can enzymatically degrade superoxide [77]. Second, oxidative stress impairs multiple components of the NO signaling pathway. Soluble guanylate cyclase (sGC), the major receptor for NO in vascular smooth muscle, shows reduced expression of its α_1_ and β_1_ subunits in chronic hypertension (in aged spontaneously hypertensive rats), consistent with a reduced vasodilator response to NO donors [91]. Downstream, cGMP-dependent protein kinase I (cGK-I), a mediator in smooth muscle relaxation, shows reduced activity, reflected by reduced phospho-VASP levels—with cGK-I expression itself preserved, indicating reduced NO/cGMP signal reaching the kinase rather than a defect in cGK-I [92]. These alterations suggest that oxidative stress disrupts NO signaling at multiple levels. Because the NO receptor sGC is itself downregulated, even with restoration of NO production, downstream signaling may remain impaired, limiting full recovery of vascular function.

Oxidative stress in prehypertension is a topic that has been specifically researched in a limited number of studies. The currently available evidence is suggestive but remains relatively sparse. Existing data show that antioxidant defense mechanisms are already compromised in the early stage of blood pressure elevation compared to normotensive controls. In one study, the activity of SOD, a key first-line antioxidant enzyme, was modestly but significantly reduced in prehypertensive individuals (9.19 ± 0.10 vs. 9.37 ± 0.29 U/mg serum protein, *p* < 0.05) [74]. Across the overall hypertensive cohort, SOD activity showed a strong negative correlation with mean arterial pressure (r = −0.773) [74]. Similar reductions were observed for catalase (8.98 ± 0.70 vs. 9.07 ± 0.07 U/mg, *p* < 0.05) and glutathione peroxidase (55.94 ± 0.30 vs. 57.09 ± 0.33 NADPH/min/mg serum protein, *p* < 0.05); across the cohort, both were inversely correlated with mean arterial pressure, GPX most strongly of the three enzymes (catalase r = −0.766; GPX r = −0.936) [74].

Together, these findings support the possibility that oxidative imbalance may already be present in prehypertension, but they also underscore the need for further studies to better define its extent, timing, and mechanistic relevance.

## 10. Temporal Sequence of Pathophysiological Events in Prehypertension

**Figure 3:**
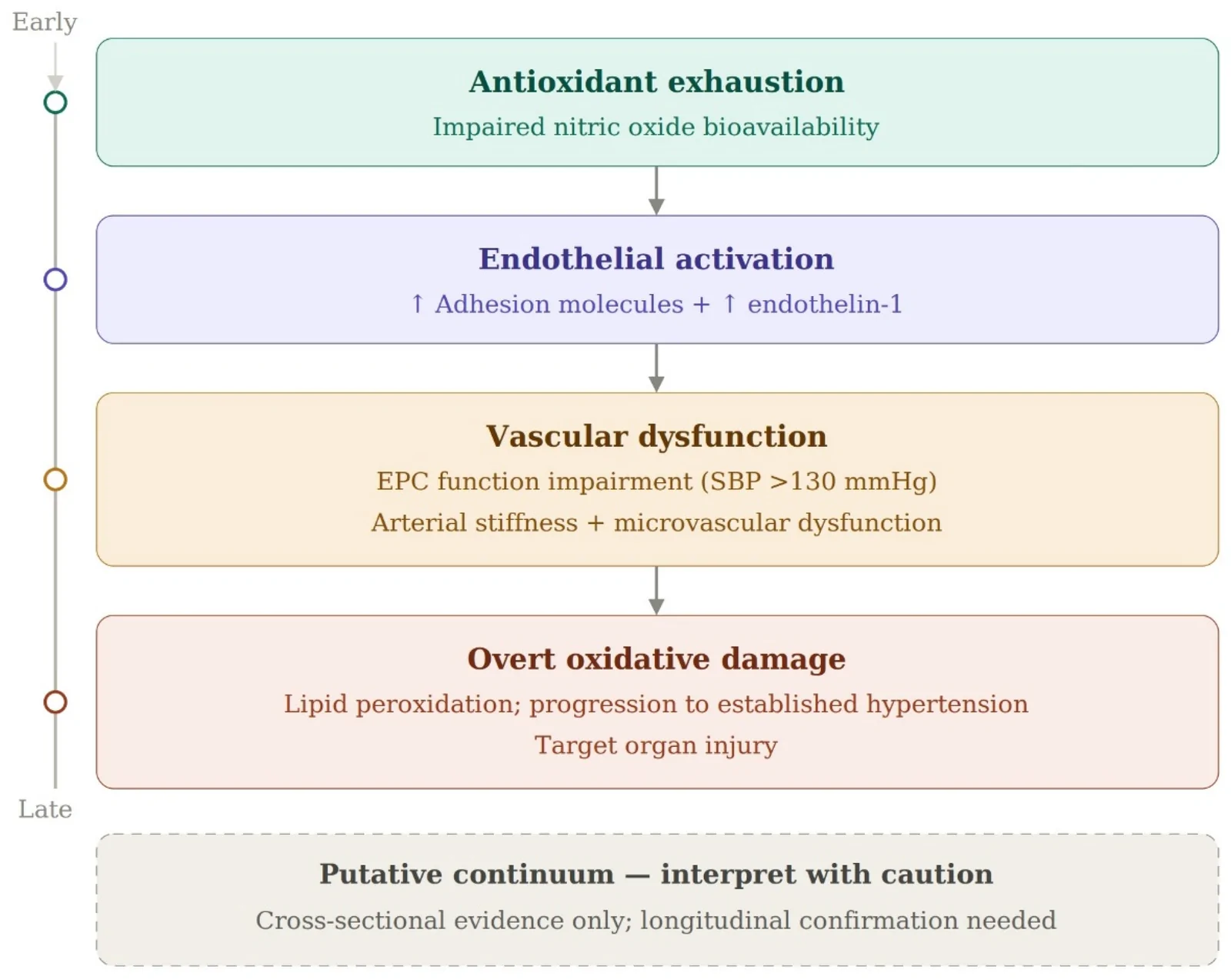
Proposed framework of oxidative–vascular progression in prehypertension: the results of available studies indicate the antioxidant exhaustion and reduced nitric oxide are among the earliest changes in the pathogenesis of the disease, which advances through endothelial activation and vascular dysfunction (impaired EPC function above 130 mmHg systolic, arterial stiffness, microvascular problems), and ends in overt oxidative damage with established hypertension and organ injury. EPC—endothelial progenitor cells; SBP—systolic blood pressure. Upward arrows (↑) indicate an increase.

Current scientific evidence does not indicate a clearly defined temporal sequence of events, but rather suggests a likely pathophysiological progression to prehypertension. Nevertheless, several potentially interconnected alterations have been reported within or near the prehypertensive blood pressure range including reduced antioxidant enzyme activity and NO-mediated vasodilation [7,74], increased circulating adhesion molecules [43], and enhanced endothelin-1 vasoconstrictor tone [50]. Impaired endothelial progenitor cell (EPC) function has also been observed, particularly at systolic blood pressure levels above 130 mmHg [93], alongside evidence of increased arterial stiffness [8] and coronary microvascular dysfunction [67]. More pronounced markers of oxidative damage, including lipid peroxidation, have been described in established hypertension [74]. Figure 3 brings these findings together in a proposed framework of vascular dysfunction across the blood pressure continuum. The connections shown are biologically plausible, but their timing and causal relationships in prehypertension remain to be established.

## 11. Research Gaps and Future Directions

It is important to emphasize that endothelial dysfunction in prehypertension has not been fully elucidated. Several important aspects of its vascular pathophysiology remain insufficiently investigated. First, most available studies were primarily focused on isolated biomarkers, individual vascular territories, or indirect functional changes in prehypertension. This heterogeneous design of available studies leaves many questions unanswered. It remains unclear whether endothelial dysfunction in prehypertension is confined to the vascular beds studied most frequently or reflects a broader systemic phenotype. Oxidative stress, impaired NO signaling, eNOS uncoupling, and altered endothelin signaling have also been implicated in dysfunction of the hepatic, pulmonary, and renal circulations, although the supporting evidence largely comes from other disease settings, including portal and pulmonary hypertension and established systemic hypertension [94,95,96]. Whether comparable changes are already present across these vascular territories in individuals with prehypertension remains unknown. Studies assessing endothelial function in multiple vascular beds within the same individuals could help address this question. Second, very few studies have simultaneously assessed multiple contributing factors—including impaired NO bioavailability, oxidative stress, endothelial activation, and defective endothelial repair—within the same individuals. Such studies are needed to better understand the complex pathophysiological processes occurring in the state of prehypertension. Third, a key unresolved issue is the temporal and mechanistic relationship between oxidative stress and endothelial dysfunction [97,98,99,100,101,102]. It is necessary to clarify the time course of the well-established influence of oxidative stress and oxidative imbalance on early vascular changes. It remains uncertain whether oxidative imbalance precedes measurable endothelial dysfunction or primarily amplifies already existing vascular injury. Clarifying this relationship could greatly contribute to the development of optimal preventive targets and interventions. With this in mind, controlled clinical studies are needed to better define the vascular profile of prehypertension. Functional vascular assessment should be integrated with biomarkers of endothelial activation and oxidative stress, as well as markers of endothelial regenerative capacity.

## 12. Conclusions

Prehypertension is not only a chronological precursor in the development of hypertension and accompanying damage but also a critical period in which vascular dysfunction already begins. Many studies show that during this period endothelial dysfunction occurs as well as impaired regenerative capacity of the vasculature. Prehypertension is also associated with increased arterial stiffness and changes in microcirculation.

Vascular injury occurs early in the course of blood pressure elevation. During this period, and before overt oxidative damage, a reduction in the amount of antioxidants in the organism and reduced NO bioavailability occurs. EPC dysfunction occurs as well as an increase in cardiovascular risk. Such findings provide support for blood pressure classification frameworks [11,103,104].

Oxidative stress may contribute to endothelial dysfunction in prehypertension through reduced NO bioavailability, eNOS uncoupling, and altered regulation of endogenous NOS inhibitors such as ADMA [34,38,77,105,106,107,108]. These pathways may interact with endothelial activation and progressive vascular dysfunction. However, direct evidence in individuals with prehypertension remains limited, and the temporal and causal relationships between oxidative imbalance, endothelial dysfunction, and blood pressure progression have yet to be established.

In conclusion, the available evidence shows that prehypertension should not be regarded solely as a borderline hemodynamic category or a statistical precursor to hypertension. Prehypertension is a biologically active and clinically relevant vascular state. Further prospective studies are needed to better define the vascular changes in prehypertension and to better clarify its prognostic significance. It is necessary to determine whether early vascular changes in prehypertension are potential targets for cardiovascular prevention with the aim of preserving cardiovascular health of the population.

## Data Availability

No new data were created or analyzed in this study. Data sharing is not applicable to this article.

## Abbreviations

The following abbreviations are used in this manuscript:

JNC 7	Seventh Report of the Joint National Committee on Prevention, Detection, Evaluation, and Treatment of High Blood Pressure
SBP	systolic blood pressure
DBP	diastolic blood pressure
ACC/AHA	American College of Cardiology/American Heart Association
ESC/ESH	European Society of Cardiology/European Society of Hypertension
NO	nitric oxide
EPC	endothelial progenitor cell
eNOS	endothelial NO synthase
BH4	tetrahydrobiopterin
BH2	dihydrobiopterin
ROS	reactive oxygen species
ADMA	asymmetric dimethylarginine
NOS	nitric oxide synthase
PRMT-1	protein-arginine methyltransferase type 1
DDAH-1	dimethylarginine dimethylaminohydrolase 1
DDAH-2	dimethylarginine dimethylaminohydrolase 2
ICAM-1	intercellular adhesion molecule-1
VCAM-1	vascular cell adhesion molecule-1
ET-1	endothelin-1
ETA	endothelin type A receptor
vWF	von Willebrand factor
NSF	N-ethylmaleimide-sensitive factor
FMD	flow-mediated dilation
CFR	coronary flow reserve
CRAE	central retinal arteriolar equivalent
PORH	post-occlusive reactive hyperemia
AT_1_R	angiotensin II receptor type 1
SOD	superoxide dismutase
sGC	soluble guanylate cyclase
cGK-I	cGMP-dependent protein kinase I
